# Genome-wide Interrogation of Protein-DNA Interactions in Mammalian Cells Using ChIPmentation

**DOI:** 10.1016/j.xpro.2020.100187

**Published:** 2020-12-04

**Authors:** Wei Xu, Ying Ye, Andrew D. Sharrocks, Wensheng Zhang, Xi Chen

**Affiliations:** 1 Department of Biology, Southern University of Science and Technology, Shenzhen, Guangdong 518055, China; 2 Cam-Su Genomic Resource Center, Soochow University, Suzhou 215123, China; 3 Faculty of Biology, Medicine and Health, University of Manchester, Oxford Rd, Manchester M13 9PL, UK

**Keywords:** Genomics, Sequencing, ChIPseq, Chromatin immunoprecipitation (ChIP)

## Abstract

Mapping the genomic locations of chromatin-associated proteins, such as transcription factors and histone modifications, is key to understanding the mechanisms of transcriptional regulation. ChIPmentation offers a simple and robust way of investigating the genomic binding sites of a protein using relatively low-input material. Here, we present a detailed protocol for the key steps that lead to a successful ChIPmentation experiment, as well as a quick analysis pipeline to examine the data.

For complete details on the use and execution of this protocol, please refer to Schmidl et al. (2015). For example data produced by this protocol, please refer to Henriksson et al. (2019) and Zhang et al. (2019).

## Highlights

• Preparation of a transposase-assisted ChIP-seq library
• A simpler and cheaper approach than traditional ChIP-seq
• A sensitive and reliable method for transcription factors and histone modifications

## Graphical Abstract

**Figure:**
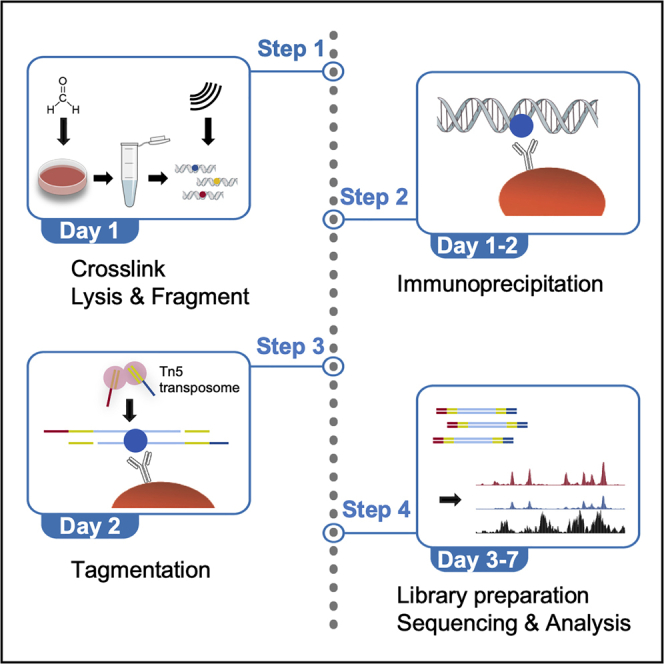

## Before You Begin

There are many existing ChIP-seq protocols with different modifications. Many of them are lengthy and difficult to carry out. We have found ChIPmentation is the simplest and the most robust to implement in a molecular biology lab, especially for people who have already had the experience with the ChIP technique. The ChIPmentation method is modular, where it contains a ChIP module and a library preparation module. Experienced researchers can just stick to their own ChIP protocol and start following the procedures described here after washing the IP (i.e., step 13). People with no previous ChIP experience are recommended to follow the exact procedures described in this protocol. It is also recommended to read the full protocol before starting in order to get a feeling about the timing and work load in each step.

Compared to other ChIP-seq methods, one advantage of ChIPmentation is its sensitivity. We routinely use 5 × 10^5^ cells to profile histone modifications and 5 × 10^6^ cells to study transcription factors. The minimum cell number required for a successful ChIPmentation experiment in our hands is 10^4^ cells for histone modifications and 10^5^ cells for transcription factors. However, it is worth noting that the number of cells required for a successful ChIPmentation experiment depends on many factors, such as the abundance of the protein/modification of interest and the efficiency of the antibody. The other major advantage of ChIPmentation is simplicity. Sequencing adapters are added by the transposase Tn5, and library PCR is performed immediately after reverse crosslinking and DNA purification. The third advantage is the cost. Only a small amount of Tn5 transposase is needed per library. Nowadays, we always use ChIPmentation even when the cell number is not a constraint, such as cell lines.

### Prepare Reagents and Buffers

**Timing: 3 h**

1. Prepare the following buffers, sterilize by using .22 μm filter units. See Materials and Equipment for buffer recipes.
  a. 100× protease inhibitor cocktail stock
  b. 11% formaldehyde
  c. 1.25 M Glycine
  d. Blocking Solution
  e. Sonication/IP Buffer
  f. RIPA Wash Buffer
  g. Low Salt Wash Buffer
  h. High Salt Wash Buffer
  i. LiCl Wash Buffer
  j. 10 mM Tris-HCl, pH 8.0
  k. 1× TE 50 mM NaCl
  l. ChIP Elution Buffer
  m. 2× Tagmentation DNA (TD) Buffer
2. Prepare oligo stocks by resuspend primers in ddH_2_O to reach 100 μM stock concentration. See Table 1 below for sequences. We order them from Sangon Biotech (Shanghai) with standard desalt purification.

**Table 1:** ChIPmentation Oligos Used for Library PCR (Illumina Nextera Index Primers)

Oligo Name	Oligo Sequence (5′ to 3′)
N701	CAAGCAGAAGACGGCATACGAGATTCGCCTTAGTCTCGTGGGCTCGG
N702	CAAGCAGAAGACGGCATACGAGATCTAGTACGGTCTCGTGGGCTCGG
N703	CAAGCAGAAGACGGCATACGAGATTTCTGCCTGTCTCGTGGGCTCGG
N704	CAAGCAGAAGACGGCATACGAGATGCTCAGGAGTCTCGTGGGCTCGG
N705	CAAGCAGAAGACGGCATACGAGATAGGAGTCCGTCTCGTGGGCTCGG
N706	CAAGCAGAAGACGGCATACGAGATCATGCCTAGTCTCGTGGGCTCGG
N707	CAAGCAGAAGACGGCATACGAGATGTAGAGAGGTCTCGTGGGCTCGG
N710	CAAGCAGAAGACGGCATACGAGATCAGCCTCGGTCTCGTGGGCTCGG
N711	CAAGCAGAAGACGGCATACGAGATTGCCTCTTGTCTCGTGGGCTCGG
N712	CAAGCAGAAGACGGCATACGAGATTCCTCTACGTCTCGTGGGCTCGG
N714	CAAGCAGAAGACGGCATACGAGATTCATGAGCGTCTCGTGGGCTCGG
S502	AATGATACGGCGACCACCGAGATCTACACCTCTCTATTCGTCGGCAGCGTC
S503	AATGATACGGCGACCACCGAGATCTACACTATCCTCTTCGTCGGCAGCGTC
S505	AATGATACGGCGACCACCGAGATCTACACGTAAGGAGTCGTCGGCAGCGTC
S506	AATGATACGGCGACCACCGAGATCTACACACTGCATATCGTCGGCAGCGTC
S507	AATGATACGGCGACCACCGAGATCTACACAAGGAGTATCGTCGGCAGCGTC
S508	AATGATACGGCGACCACCGAGATCTACACCTAAGCCTTCGTCGGCAGCGTC
S510	AATGATACGGCGACCACCGAGATCTACACCGTCTAATTCGTCGGCAGCGTC
S511	AATGATACGGCGACCACCGAGATCTACACTCTCTCCGTCGTCGGCAGCGTC

### Sonication Test and Antibody Test I

**Timing: 3 days or more**

***Note:*** It is very important to find the right condition for sonication and a good antibody for the immunoprecipitation of the protein of your interest. In general, one can refer to the ENCODE and modENCODE guidelines (Landt et al., 2012). This section describes some extra details on how to check sonication and perform an initial test on antibodies. Sonication serves two purposes: to solubilize chromatin and to fragment DNA to a size range that is suitable for next generation sequencing. However, during the sonication process, we found the epitopes of some proteins can be destroyed as well. The main purpose here is to find a balance between getting the DNA to the right range and maintain the protein integrity at the same time. This is the most variable process in the entire protocol, because sonication is highly dependent on the machine in use. We have successful experience using both probe sonicators and water bath sonicators.

3. Follow steps 1–3 and 5–6 from the Step-by-Step Method Details. We prepare cells that are enough for 4–5 aliquots (5 × 10^6^ per aliquot) for the sonication test.

4. After resuspending cell pellet at a concentration of 5 × 10^6^ cells per 300 μL Sonication/IP Buffer (i.e., step 6):
  ***Note:*** both the cell concentration and the volume can influence sonication results. We routinely used 300 μL Sonication/IP Buffer to resuspend cells from 10^5^ to 5 × 10^6^. When more than 5 × 10^6^ cells are used, we scale up the volume to maintain a concentration of 5 × 10^6^ per 300 μL volume. If the final volume exceeds the recommendation of the sonicator, make aliquots to perform sonication.
  a. Take out 25 μL lysate and mix with 75 μL ChIP Elution Buffer and 1 μL Proteinase K (20 mg/mL). Leave the reaction on a thermomixer at 65°C, with shaking at 1,400 rpm for at least 6 h (or overnight, 12–16 h) for the reverse crosslink. This is the no sonication DNA input.
  b. Take out 32 μL lysate and mix with 8 μL 5× SDS Loading Buffer. Boil at 99°C for 10 min. This is the no sonication protein input.

5. If using a water bath sonicator, aliquot 300 μL lysate into 1.5 mL Eppendorf tubes (or other tubes required by the sonicator manufacturer). Perform a sonication time course with a recommended ON/OFF cycle setting. For example, if using a Bioruptor Pico, a time course of 2, 4, 6, 8 min with 30 s ON/30 s OFF can be used for an initial trial. At the end of each time course:
  a. Take out 25 μL lysate from an aliquot and mix with 75 μL ChIP Elution Buffer and 1 μL Proteinase K (20 mg/mL). Leave the reaction on a thermomixer at 65°C, with shaking at 1,400 rpm for at least 6 h (or overnight, 12–20 h) for the reverse crosslink. This is for the DNA size check.
  b. Take out 32 μL lysate and mix with 8 μL 5× SDS Loading Buffer. Boil at 99°C for 10 min. This is for the protein check.

***Note:*** When using the Bioruptor water bath sonicator, the maximum recommended volume in a 1.5 mL Eppendorf tube is 300 μL. Therefore, it is not possible to just use one tube for the entire time course due the volume needed for the DNA and protein analysis. We normally prepare multiple tubes, one for each time point.

**Figure 1:**
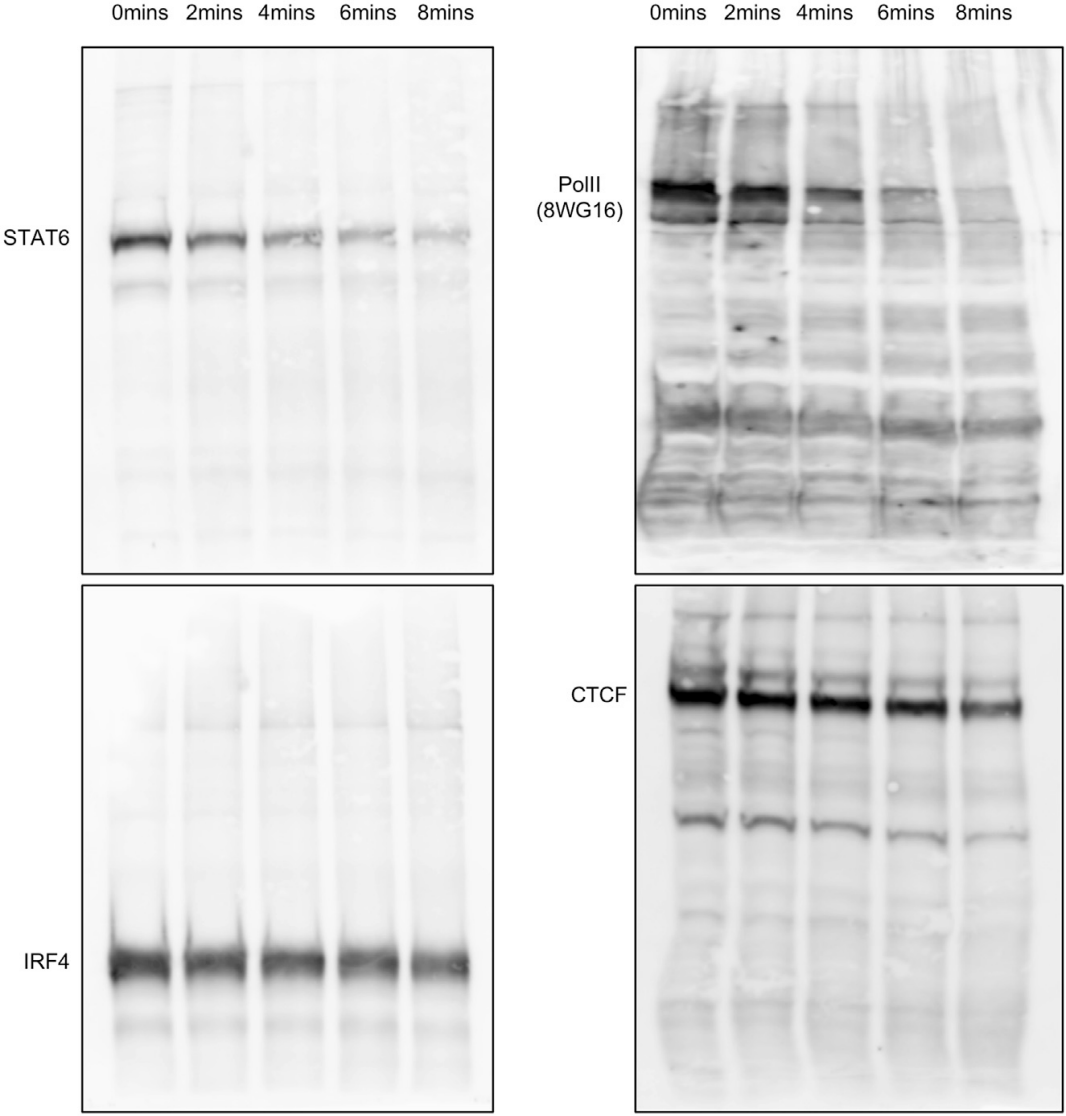
Checking the Antibody and Protein Integrity before and during the Sonication Time Course The sonication time and antibodies in use are indicated in the figure.

6. Analyze all the protein samples using western blot with the antibody of your choice. We include in the Key Resources Table the antibodies used in this particular protocol, but the antibodies will depend on the factors of interest. Four examples are shown in Figure 1.

**CRITICAL:** This is the initial test of the antibody and protein integrity. There are two things to check here. First, in the no sonication input (0 min), a single (or major) clear band around the predicted size of the protein of interest is present. In Figure 1, all four antibodies satisfy this standard. Second, the protein of interest remains detectable during sonication. In Figure 1, different proteins perform differently. STAT6 and Pol II start to become less visible after 4 min, while IRF4 and CTCF remain relatively stable until 8 min. See the next step to decide which condition to use. For some lowly expressed proteins, more concentrated cell lysate needs to be used to visualize them on the western blot.

**Figure 2:**
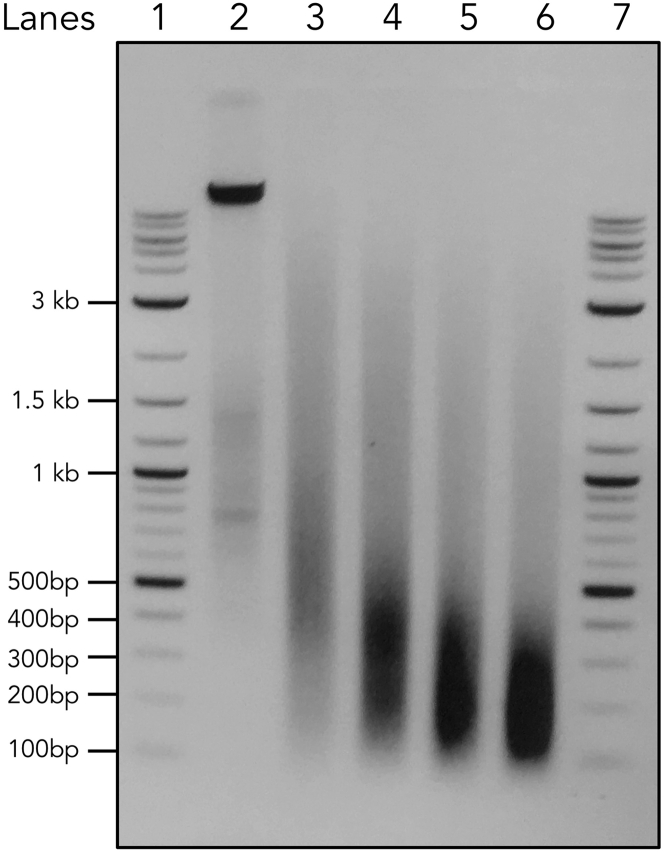
DNA Size Distribution during the Sonication Time Course Lanes 1 and 7 are NEB 2-log DNA ladder. Lane 2 is the input DNA before sonication. Lanes 3–6 are purified input DNA sonicated with 2, 4, 6, 8 min.

7. Purify all the DNA samples after the reverse crosslink using the Qiagen minElute PCR Purification Kit. Determine the DNA concentration using a Nanodrop. Run equal amount of DNA (we routinely use 500–1,000 ng) on a 1.5% Agarose gel. An example is shown in Figure 2.

**CRITICAL:** Normally one should choose the minimum sonication time that gives rise to the ideal range (100–500 bp). However, the results from the western blot and DNA gel need to be considered together to make a decision. An earliest condition where the DNA is sheared and the protein is still detectable should be chosen. ChIPmentation will use Tn5 to “cut and paste” the sequencing adapters to the DNA after the immunoprecipitation, which results in the fragmentation of DNA for a second time. Therefore, it is generally okay to have slightly larger DNA fragments comparing to the traditional ChIP-seq method at this stage. As long as the majority of the DNA is below 1,000 bp and there is no clear band above 1,000 bp, we accept the condition. In this case, Lane 3 is chosen. See Troubleshooting 1 for some tips.

### Antibody Test II

**Timing: 3 days or more**

In the previous section, the sonication condition is determined and whether the antibody is able to detect the protein of interest is also tested. In this section, procedures are described to test if the antibody can immunoprecipitate the protein of interest after formaldehyde crosslinking. The success of antibody in this test does not necessarily guarantee a successful ChIPmentation experiment, but it provides some useful information about the antibody and the immunoprecipitation condition.

**Figure 3:**
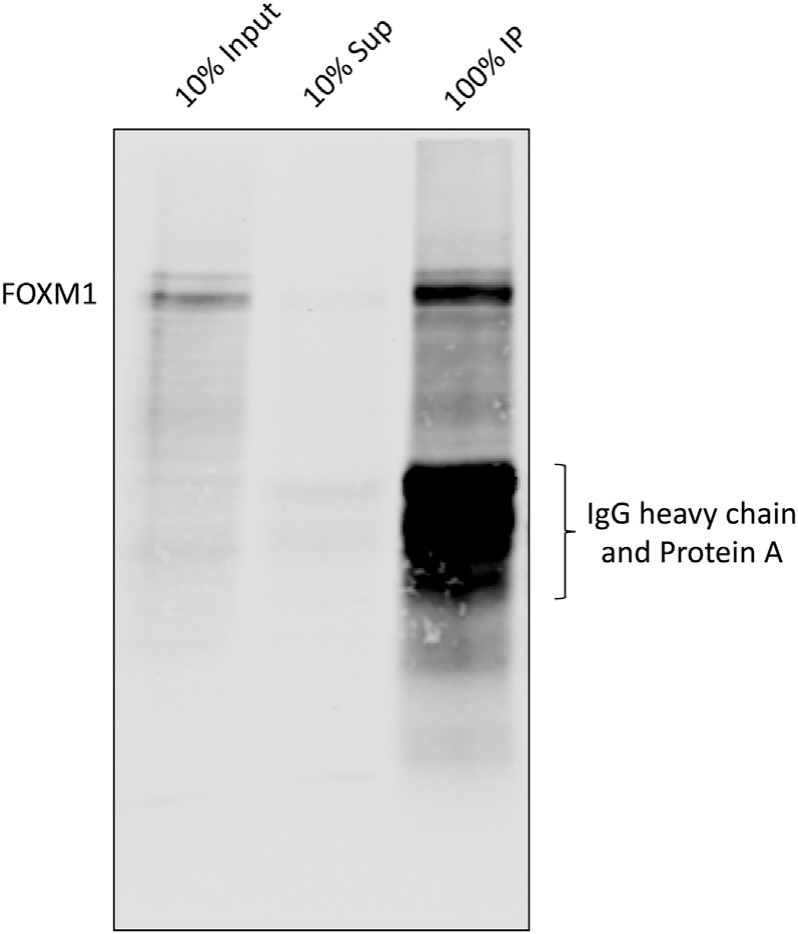
ChIP-Western Results Demonstrating the FOXM1 Antibody Is Able to Immunoprecipitate the Protein of Interest (i.e., FOXM1) after the Whole ChIP Procedure The identities of the samples and bands are indicated in the figure.

8. Follow steps 1–16 from the Step-by-Step Method Details.
  a. At the step 6, take 32 μL lysate and mix with 8 μL 5× SDS Loading Buffer. Boil at 99°C for 10 min. This is the input sample and can be stored in −20°C and will be used in the next day.
  b. At the step 8, before washing, put the immunoprecipitation on the magnetic stand, and take out 32 μL lysate and mix with 8 μL 5× SDS Loading Buffer. Boil at 99°C for 10 min. This is the supernatant.
  c. After the step 16, instead of adding tagmentation mix, add 40 μL 1× SDS Loading Buffer (diluted in Sonication/IP Buffer from 5× SDS Loading Buffer) to the beads. Boil at 99°C for 10 min. This is the IP sample.
9. Analyze all the samples (input, supernatant and IP) using western blot with the same antibody. An example using an anti-FOXM1 antibody is shown in Figure 3.

**Figure 4:**
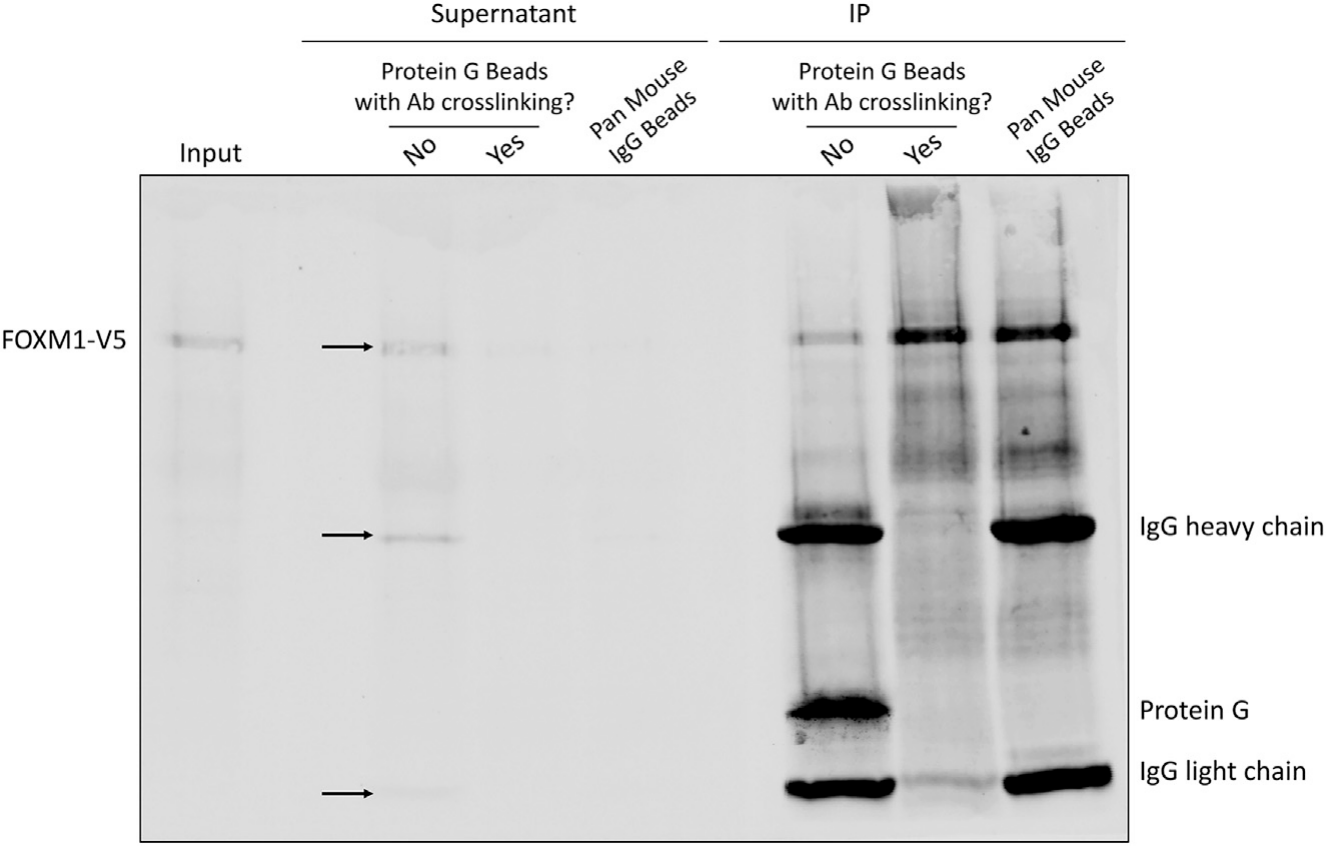
ChIP-Western Results with a Mouse Monoclonal V5 Antibody in a Cell Line Stably Expressing V5-Tagged FOXM1 Note the presence (indicated by arrows) of FOXM1-V5, the heavy and light chains of IgG in the supernatant when using Protein G Dynabeads without crosslinking the antibody to the beads. All the aforementioned bands disappear when the antibody is crosslinked to the Protein G Dynabeads or Pan Mouse IgG Dynabeads are used.

**CRITICAL:** In Figure 3 shown above, 1 μg of anti-FOXM1 antibody was used to immunoprecipitate the chromatin from 5 × 10^6^ U2OS cells. When a different antibody is used, different ratios of antibody:chromatin input need to be tested to find a good condition to achieve the result in Figure 3. There are three things to check here. First, a single (or major) clear band around the predicted size of the protein of interest should be present in the input lane. Second, a single (or major) clear band at the same size as the input should be present in the IP lane. One can roughly calculate the IP efficiency based on the intensities of the protein bands, but we generally found the efficiencies of many transcription factor antibodies are low. Nevertheless, they produce successful ChIPmentation results. Third, no or very minimum of the protein of interest or IgG band is visible in the supernatant lane. If the protein of interest is clearly visible in the supernatant, it means the antibody fails to pull down the protein. Whenever this happens, we also observe IgG bands in the supernatant at the same time. This mostly happens with some mouse IgG antibodies that have low affinity to Protein G. We have found the most efficient ways of solving this problem is to either chemically crosslink the antibody to the beads using DSP (or the like) or change to a different type of beads, such as the Pan Mouse IgG Dynabeads. An example using a mouse monoclonal anti-V5 antibody to pull down V5-tagged FOXM1 is shown in Figure 4 to demonstrate this critical point.

## Key Resources Table

**Table:** 

REAGENT or RESOURCE	SOURCE	IDENTIFIER
**Antibodies**		

Stat6 Antibody (M-20)	Santa Cruz Biotechnology	sc-981
FOXM1 Antibody (C-20)	Santa Cruz Biotechnology	sc-502
IRF-4 Antibody (M-17)	Santa Cruz Biotechnology	sc-6059
Anti-RNA polymerase II CTD antibody (8WG16)	Abcam	ab817
Anti-CTCF Antibody	Millipore	Cat# 07-729
Anti-V5 antibody, Mouse monoclonal	Sigma	V8012

**Chemicals, Peptides, and Recombinant Proteins**		

10× PBS, pH 7.4	Thermo Fisher	AM9624
BSA	Sigma	V900933
1 M HEPES, pH 7.5	Thermo Fisher	Cat# 15630106
5 M NaCl	Sigma	S6546
0.5 M EDTA, pH 8.0	Thermo Fisher	AM9260G
0.5 M EGTA, pH 8.0	Sigma	E3889
Triton X-100	Sigma	Cat# 93443
Sodium Deoxycholate (DOC)	Sigma	Cat# 30970
10% Sodium Dodecyl Sulfate (SDS)	Thermo Fisher	Cat# 15553027
1 M Tris-HCl, pH 8.0	Thermo Fisher	Cat# 15568025
1 M MgCl_2_	Thermo Fisher	AM9530G
IGEPAL CA-630	Sigma	I8896
LiCl	Sigma	Cat# 62476
Proteinase K	Thermo Fisher	AM2546
Protease Inhibitor Cocktail	Roche	Cat# 1697498
N,N-Dimethylformamide	Sigma	D4551
37% Formaldehyde solution	Sigma	Cat# 252549
Glycine	Sigma	G8790
20× EvaGreen	Biotium	Cat# 31000-T
NEBNext High-Fidelity 2× PCR Master Mix	NEB	M0541
Dynabeads Protein A for Immunoprecipitation	Thermo Fisher	10001D
Dynabeads Protein G for Immunoprecipitation	Thermo Fisher	10003D
Dynabeads Pan Mouse IgG	Thermo Fisher	11041
AmpureXP for PCR Purification	Beckman Coulter	A63881
VAHTS DNA Clean Beads	Vazyme Biotech	N411

**Critical Commercial Assays**		

Illumina Tagment DNA TDE1 Enzyme and Buffer kit	Illumina	Cat# 20034197
Fapon Tnp Library Prep Kit for Illumina	Fapon Biotech	NK001
MinElute PCR Purification Kit	Qiagen	Cat# 28004
Agilent High Sensitivity DNA Kit	Agilent	Cat# 5067-4626

**Deposited Data**		

ChIP-seq data	(Zhang et al., 2019)	ArrayExpress: E-MTAB-6165

**Software and Algorithms**		

Fastp	(Chen et al., 2018)	https://github.com/OpenGene/fastp
HISAT2	(Kim et al., 2019)	https://github.com/DaehwanKimLab/hisat2
Picard	https://github.com/broadinstitute/picard	http://broadinstitute.github.io/picard/
MACS2	(Zhang et al., 2008)	https://github.com/macs3-project/MACS
bdg2bw	https://gist.github.com/taoliu/2469050	https://gist.github.com/taoliu/2469050
fetchChromSizes	(Kent et al., 2002)	http://hgdownload.soe.ucsc.edu/admin/exe/
bedClip	(Kent et al., 2002)	http://hgdownload.soe.ucsc.edu/admin/exe/
bedGraphToBigWig	(Kent et al., 2002)	http://hgdownload.soe.ucsc.edu/admin/exe/
Samtools	(Li et al., 2009)	http://www.htslib.org/

## Materials and Equipment

### Buffers

***Alternatives:*** all the chemicals and solutions listed in the Key Resources Table can be purchased from different suppliers for your own convenience, provided they are all molecular biology grade.

**Table:** 100× Protease Inhibitor Cocktail stock (aliquot and store at −20°C)

Reagent	Final Concentration	Amount
Protease Inhibitor Cocktail	100×	1 tablet∗
ddH_2_O	n/a	1 mL
**Total**	**n/a**	**1 mL**

∗If Protease Inhibitor cocktail solution is purchased, ignore this table and use the solution according the supplier’s recommendation.

**Table:** 11% formaldehyde solution (freshly prepared each time, and prepare just enough for the experiment to reduce the waste)

Reagent	Final Concentration	Amount
1 M HEPES, pH 7.5	50 mM	0.5 mL
5 M NaCl	100 mM	0.2 mL
0.5 M EDTA, pH 8.0	1 mM	20 μL
0.5 M EGTA, pH 8.0	0.5 mM	10 μL
37% formaldehyde	11%	2.97 mL
ddH_2_O	n/a	6.3 mL
**Total**	**n/a**	**10 mL**

**CRITICAL:** formaldehyde is highly toxic, should be used with proper personal protective equipment in a fume hood. Waste should discarded according to local regulations for hazardous waste.

**Table:** 1.25 M Glycine (store at room temperature: 20°C–25°C)

Reagent	Final Concentration	Amount
Glycine	1.25 M	9.38 g
ddH_2_O	n/a	Add to 100 mL
**Total**	**n/a**	**100 mL**

**Table:** Blocking Solution (store at 4°C)

Reagent	Final Concentration	Amount
10× PBS (pH 7.4)	1×	10 mL
BSA	0.5%	0.5 g
ddH_2_O	n/a	Add to 100 mL
**Total**	**n/a**	**100 mL**

**Table:** Sonication/IP Buffer (store at 4°C)

Reagent	Final Concentration	Amount
1 M HEPES, pH 7.5	50 mM	5 mL
5 M NaCl	140 mM	2.8 mL
0.5 M EDTA, pH 8.0	1 mM	0.2 mL
10% Triton X-100	1%	10 mL
10% DOC	0.1%	1 mL
10% SDS	0.1%	1 mL
ddH_2_O	n/a	80 mL
**Total**	**n/a**	**100 mL**

**Table:** RIPA Wash Buffer (store at 4°C)

Reagent	Final Concentration	Amount
1 M Tris-HCl, pH 8.0	50 mM	5 mL
5 M NaCl	150 mM	3 mL
0.5 M EDTA, pH 8.0	2 mM	0.4 mL
10% IGEPAL-CA630	1%	10 mL
10% DOC	0.1%	1 mL
10% SDS	0.1%	1 mL
ddH_2_O	n/a	79.6 mL
**Total**	**n/a**	**100 mL**

**Table:** Low Salt Wash Buffer (store at 4°C)

Reagent	Final Concentration	Amount
1 M Tris-HCl, pH 8.0	20 mM	2 mL
5 M NaCl	150 mM	3 mL
0.5 M EDTA, pH 8.0	2 mM	0.4 mL
10% Triton X-100	1%	10 mL
10% SDS	0.1%	1 mL
ddH_2_O	n/a	83.6 mL
**Total**	**n/a**	**100 mL**

**Table:** High Salt Wash Buffer (store at 4°C)

Reagent	Final Concentration	Amount
1 M Tris-HCl, pH 8.0	20 mM	2 mL
5 M NaCl	500 mM	10 mL
0.5 M EDTA, pH 8.0	2 mM	0.4 mL
10% Triton X-100	1%	10 mL
10% SDS	0.1%	1 mL
ddH_2_O	n/a	76.6 mL
**Total**	**n/a**	**100 mL**

**Table:** LiCl Wash Buffer (store at 4°C)

Reagent	Final Concentration	Amount
1 M Tris-HCl, pH 8.0	10 mM	1 mL
5 M LiCl	250 mM	5 mL
0.5 M EDTA, pH 8.0	1 mM	0.2 mL
10% IGEPAL CA-630	1%	10 mL
10% DOC	0.5%	5 mL
ddH_2_O	n/a	78.8 mL
**Total**	**n/a**	**100 mL**

**Table:** 10 mM Tris-HCl, pH 8.0 (store at 4°C)

Reagent	Final Concentration	Amount
1 M Tris-HCl, pH 8.0	10 mM	1 mL
ddH_2_O	n/a	99 mL
**Total**	**n/a**	**100 mL**

**Table:** 1× TE + 50 mM NaCl (store at 4°C)

Reagent	Final Concentration	Amount
1 M Tris-HCl, pH 8.0	10 mM	1 mL
5 M NaCl	50 mM	1 mL
0.5 M EDTA, pH 8.0	1 mM	0.2 mL
ddH_2_O	n/a	97.8 mL
**Total**	**n/a**	**100 mL**

**Table:** 2× Tagmentation DNA (TD) Buffer (store at −20°C and discard after one or two months)

Reagent	Final Concentration	Amount
1 M Tris-HCl, pH 8.0	20 mM	20 μL
1 M MgCl_2_	10 mM	10 μL
N,N-Dimethylformamide	20%	200 μL
ddH_2_O	n/a	770 μL
**Total**	**n/a**	**1 mL**

**Table:** ChIP Elution Buffer (store at room temperature: 20°C–25°C)

Reagent	Final Concentration	Amount
1 M Tris-HCl, pH 8.0	50 mM	50 μL
0.5 M EDTA, pH 8.0	10 mM	20 μL
10% SDS	1%	100 μL
ddH_2_O	n/a	830 μL
**Total**	**n/a**	**1 mL**

### Equipment

Sonicator: Diagenode Bioruptor Pico

***Alternatives:*** Other systems such as Bioruptor Plus, Covaris, and probe sonicators.

Magnet: DynaMag-2

***Alternatives:*** Any magnet with tube racks.

qPCR machine: QuantStudio1 Real-Time PCR Machine.

***Alternatives:*** Any qPCR machine.

Library QC: Agilent Bioanalyzer 2100

***Alternatives:*** Other systems such as Agilent TapeStation, Fragment Analyzer, Caliper LabChip GX.

## Step-By-Step Method Details

### Fixation of Cells and Binding Antibodies to Dynabeads

**Timing: 6 h to overnight**

This section fixes cultured cells with formaldehyde, which preserves protein-DNA and protein-protein interactions. In addition, antibody-beads complex is prepared for immunoprecipitation. When working with tissues, use an appropriate method to dissociate the tissue into single-cell suspension and start the protocol from step 2b. We normally use 5 × 10^5^ cells for profiling histone modifications and 5 × 10^6^ cells for transcription factors.

1. Add 1/10 volume of 11% formaldehyde solution directly to the culture media in plates. Swirl briefly and incubate at room temperature (20°C–25°C) for 10 min.
2. Add 1/10 volume of 1.25 M Glycine, swirl briefly and incubate at room temperature (20°C–25°C) for 5 min to stop formaldehyde crosslinking.
  a. For adherent cells, remove all liquid in the plate, and rinse with ice-cold 1× PBS (pH 7.4) twice. Collect cells into 1 mL ice-cold 1× PBS (pH 7.4) supplied with 1% Fetal Bovine Serum (FBS) using a cell scraper, and transfer to 1.5 mL Eppendorf tubes. Spin at 4°C for 5 min at 1,000 × *g*, and discard supernatant.
  b. For suspension cells:
    i. Transfer enough cells into either Eppendorf tubes or conical tubes. If no serum is in the culture media, add 1/100 volume of FBS.
    ii. Spin at 4°C for 5 min at 1,000 × *g*, and discard supernatant.
    iii. Resuspend cell pellet with the same amount of ice-cold 1× PBS (pH 7.4), spin at 4°C for 5 min at 1,000 × *g*, and discard supernatant. Repeat once.

**CRITICAL:** The addition of FBS during those steps help reduce the loss of cells, especially when the cell number is small.

3. Cell pellets can be snap freezed in liquid nitrogen and store in −80°C for at least 6 months. Or cell pellets can be used immediately. See the next section.
4. Bind antibodies to Dynabeads. We use 10 μL Dynabeads and 1 μg antibody per ChIP. Protein A or G Dynabeads, or Pan Mouse IgG Dynabeads are chosen based on the primary antibody being used and the results from the previous section. Prepare each ChIP individually in different Eppendorf tubes.
  a. Mix 10 μL Dynabeads with 500 μL Blocking Solution in the tube, and collect beads using DynaMag-2. Allow beads to set at the side of the tube. Invert twice or three times to collect beads at the tube cap. No need to centrifuge. Remove the supernatant with an aspirator.
  b. Add 500 μL Blocking Solution to wash the beads. This can be done by removing the rack from the magnet and inverting the rack with tubes still in place for 20 times or until the beads are evenly distributed in the Blocking Solution.
  c. Repeat the above wash twice, to reach a total of three washes.
  d. Resuspend the washed beads in 250 μL Blocking Solution, add 1 μg antibody, and put on a rotator at 4°C for at least 6 h or overnight (12–20 h).

### Sonication and Immunoprecipitation of Chromatin

**Timing: 1 h hands-on time and overnight immunoprecipitation**

This section describes the procedures to solubilize and break chromatin into appropriate size range, and use the specific antibody to immunoprecipitate the DNA bound by the protein of interest.

5. If using the frozen pellet from the steps described above, take the pellet from −80°C and thaw on ice.
6. Resuspend the pellet of appropriate cell numbers in 300 μL Sonication/IP Buffer with freshly added 1× protease inhibitor cocktails, and sonicate on a Bioruptor Pico (or the alternatives) for an appropriate number of cycles based on the results from Before You Begin section.
7. Incubate the chromatin with antibody-beads complex:
  a. Centrifuge the sonicated chromatin at 16,000 × *g* at 4°C for 10 min.
  b. During the 10-min centrifugation time, wash the antibody-beads complex from step 4d three times with 500 μL Blocking Solution in the same way as described in steps 4a and 4b.
  c. Save 2 μL supernatant from step 7a, and store in −20°C as the input sample, and transfer the rest supernatant to the washed antibody-beads complex. Incubate overnight (12–20 h) at 4°C on a rotator.

***Note:*** There should be very tiny or no visible pellet after the centrifugation at step 7a.

### Wash Beads, Tagmentation on Beads, and Reverse Crosslinking

**Timing: 1 h hands-on time and 6 h to overnight reverse crosslinking**

This section describes the procedures in to wash the immunoprecipitation and add sequencing adapters via tagmentation by Tn5. Then the crosslink is reversed by heating at 65°C. All wash steps are done at the bench with wash buffers kept on ice.

8. Put the immunoprecipitation on DynaMag-2 to collect the beads at the side of the tube. Invert twice or three times to collect beads at the tube cap. No need to centrifuge.
9. Remove the supernatant, and the buffer at the cap using an aspirator or a pipette.
10. Wash once with 500 μL RIPA Wash Buffer. This can be done by removing the tube rack from the magnet, add the buffer and invert by hand with the tube still on the rack for 15–20 times or until the beads are evenly distributed in the buffer.
11. Wash once with 500 μL Low Salt Wash Buffer.
12. Wash once with 500 μL High Salt Wash Buffer.
13. Wash once with 500 μL LiCl Wash buffer.
14. Wash twice with 500 μL 10 mM Tris-HCl, pH 8.0.

***Note:*** The washes from step 11 to 14 are performed in the same way as described in step 10.

**CRITICAL:** During the wash in step 14, the beads will not attach to the magnet very tightly. Therefore, DO NOT use the aspirator to remove the buffer. Instead, use a pipette to remove the buffer carefully.

15. Collect the beads to the bottom of the tube by a brief centrifugation at 100 × *g* for 30 s.
16. Put the tube on DynaMag-2 and remove trace of Tris-HCl.
17. Resuspend the beads thoroughly with 30 μL tagmentation mix, which consists of 15 μL 2× TD Buffer + 14 μL ddH_2_O + 1 μL Tn5. The Tn5 can be from either the Illumina Tagment DNA TDE1 Enzyme and Buffer kit or the Fapon Tnp Library Prep Kit for Illumina. You only need one kit, not both.
18. Take the 2 μL input sample from −20°C, and mix with 30 μL tagmentation mix (the same as above).
19. Put both the IP and input samples on the thermomixer to incubate at 37°C for 5 min with 800 rpm shaking.

**Figure 5:**
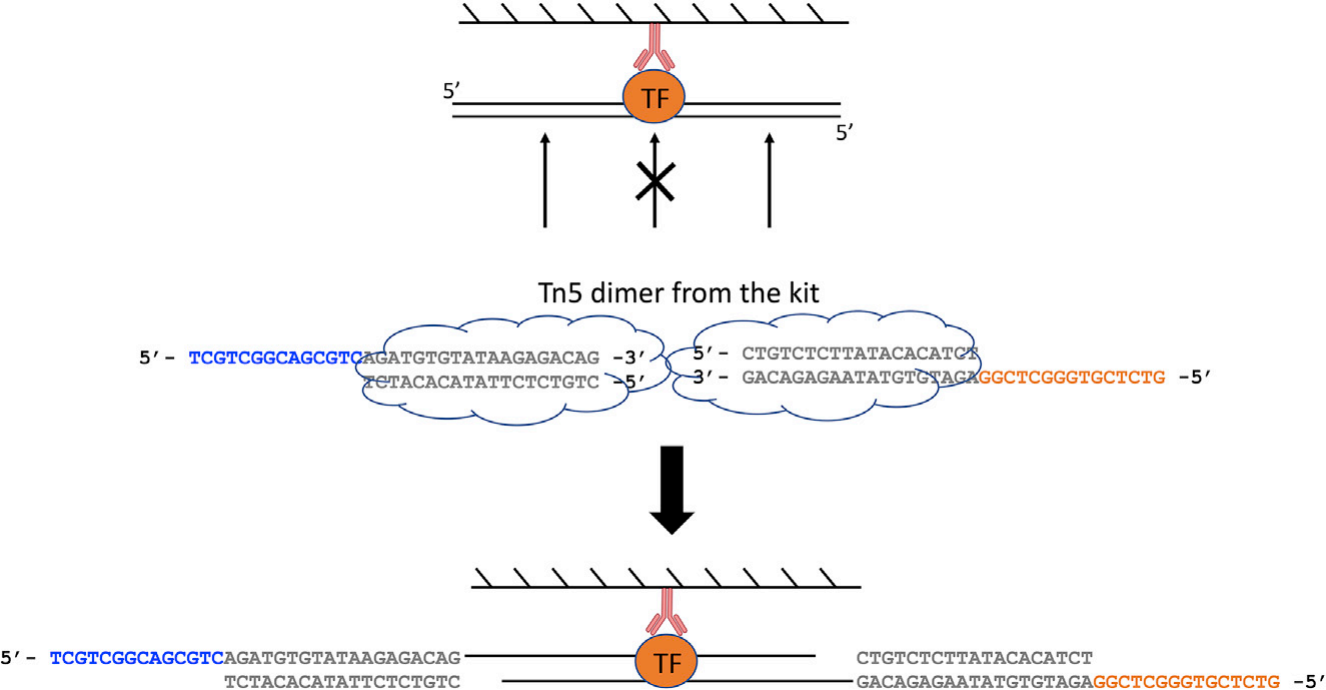
Schematic View of the Tagmentation Process on the Beads

**CRITICAL:** This incubation step allows Tn5 to add sequencing adapters to the immunoprecipitated DNA. At this stage, the DNA is still bound by the protein which protects the DNA from being cut by Tn5. Therefore, you do not need to worry about over-tagmentation. We used 1 μL for easy pipetting regardless of the cell number used here. What happens in the tube is shown in Figure 5.

20. Stop the tagmentation reaction:
  a. For input samples, directly add 70 μL ChIP Elution Buffer, add 1 μL of Proteinase K (20 mg/mL) and leave at 65°C on a thermomixer with 1,400 rpm shaking for at least 6 h or overnight (12–20 h).
  b. For IP samples, wash beads with 500 μL Low Salt Wash Buffer twice, then with 500 μL 1× TE 50 mM NaCl once. Perform the wash in the same way as described in step 10.
  c. Briefly centrifuge the IP samples to collect beads at the bottom of the tube. Put the tubes to DynaMag-2 and remove trace of 1× TE 50 mM NaCl.
  d. Add 100 μL ChIP Elution Buffer to the beads, add 1 μL Proteinase K (20 mg/mL) and briefly vortex until the beads become homogeneous.
  e. Leave all samples at 65°C on a thermomixer with 1,400 rpm shaking for at least 6 h or overnight (12–20 h).

**CRITICAL:** In step 20c, the beads will not attach to the magnet very tightly. Therefore, DO NOT use the aspirator to remove the buffer. Instead, use a pipette to remove the buffer carefully. In step 20d, take care not to vortex the beads to the cap.

### DNA Purification and Library Preparation

**Timing: 2 h**

**Figure 6:**
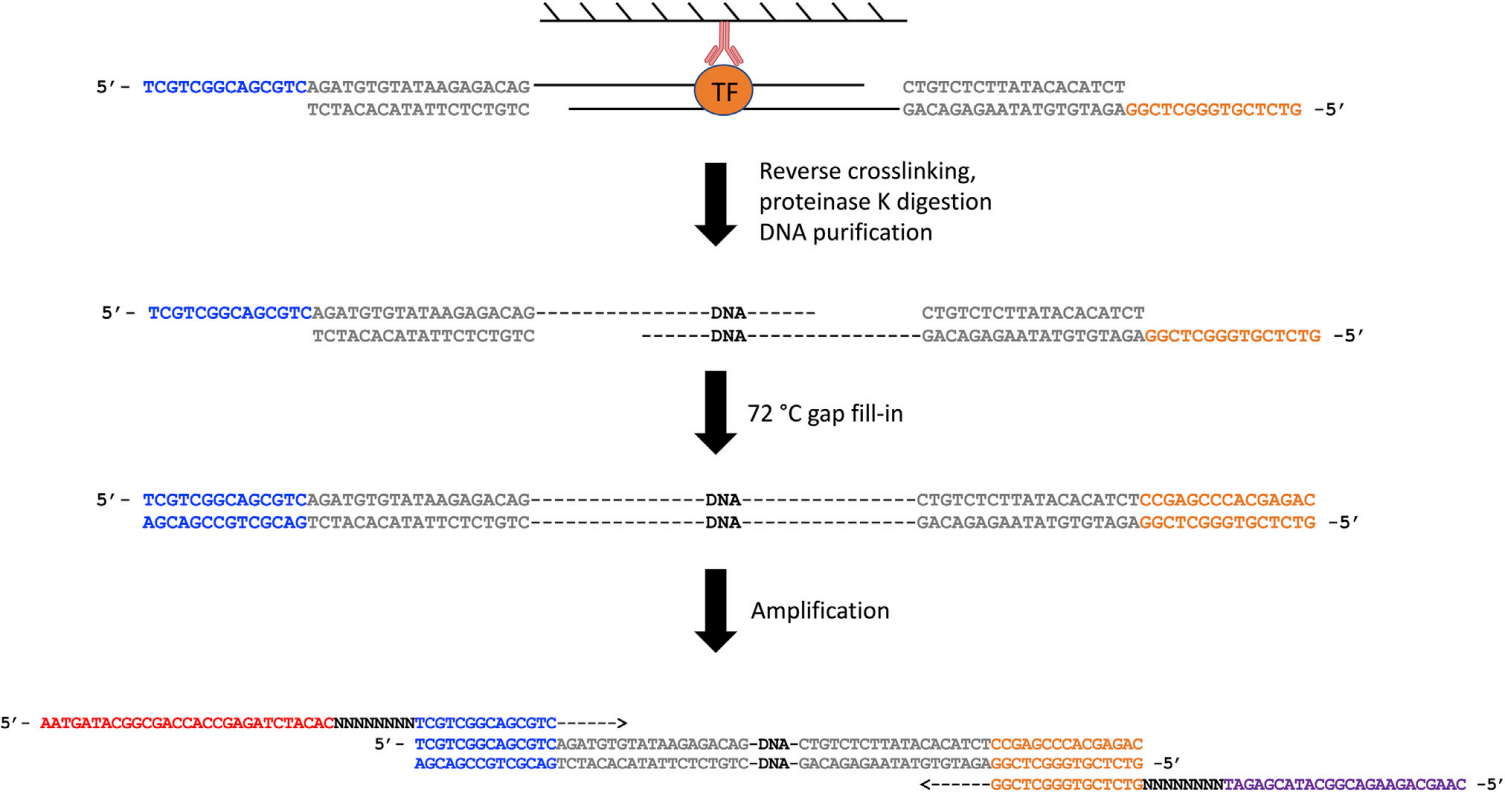
Schematic View of Steps of DNA Purification and Library Purification

This section describes the procedures of DNA purification and library preparation. See Figure 6 below for the schematic view of this section with PCR details.

21. Purify DNA from both input and IP samples using the Qiagen minElute PCR Purification Kit according to manufacturer’s instructions. Elute the DNA in 11 μL Elution Buffer from the kit twice, which generally yields 20 μL DNA.

***Note:*** There is no need to quantify the DNA concentration at this stage. Use all for the next step.

22. Setup the library PCR reaction per sample as follows:

**Table:** 

Reagent	Volume
Purified DNA from step 21	20 μL
10 μM S5xx Primer	2.5 μL
10 μM N7xx Primer	2.5 μL
NEBNext High-Fidelity 2× PCR Master Mix	25 μL
**Total**	**50 μL**

23. Run a pre-amplification PCR using the following condition:

**Table:** 

Pre-Amplification PCR Cycling Conditions			
Steps	Temperature	Time	Cycles
Gap fill-in	72°C	5 min	1
Initial Denaturation	98°C	1 min	1
Denaturation	98°C	10 s	4 cycles
Annealing	63°C	30 s	
Extension	72°C	20 s	
Hold	10°C	Forever

***Note:*** The combination of S5xx and N7xx primers identifies a sample. Therefore, different samples should use different combinations of S5xx and N7xx primers. If you do not have many samples, it is recommended to use different N7xx primers, because the index in the N7xx primer is sequenced first on an Illumina machine.

**CRITICAL:** Since the tagmentation process creates 9-bp gaps (see Figure 5), the first step in the PCR should be 72°C to allow the polymerase to fill in the gaps.

24. After the pre-amplification, take out 9 μL of the reaction, and mix with 1 μL 10× EvaGreen and perform a qPCR analysis to decide the optimal cycle number. Leave the rest 41 μL reaction on ice.
25. Use the following cycling condition to perform a qPCR analysis, and monitor the amplification curve in linear scale.

**Table:** 

qPCR Cycling Conditions			
Steps	Temperature	Time	Cycles
Initial Denaturation	98°C	1 min	1
Denaturation	98°C	10 s	35 cycles
Annealing	63°C	30 s	
Extension	72°C	20 s (acquire data)

**Figure 7:**
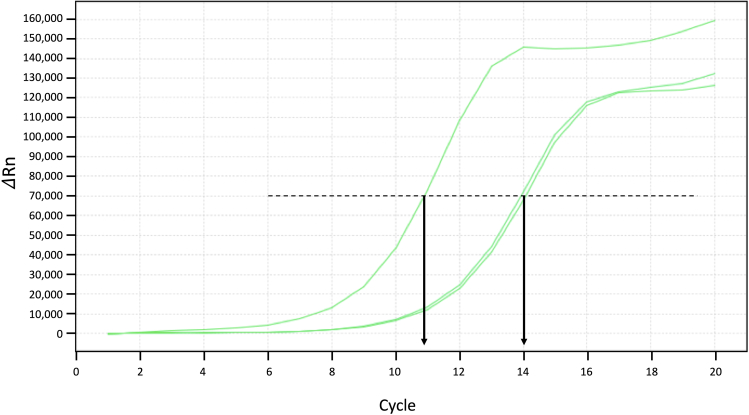
Amplification Plot of qPCR

***Note:*** The cycle number should be chosen at the exponential phase, before reaching saturation.

26. Determine the cycle number N, where the amplification curve reach half way of saturation. In the examples shown in the Figure 7 below, N = 11, 14, and 14 for the three different samples.

27. Once the optimal cycle number is decided, amplify the rest 41 μL reaction for a further of N cycles, using the following condition:

**Table:** 

Post-Amplification PCR Cycling Conditions			
Steps	Temperature	Time	Cycles
Initial Denaturation	98°C	1 min	1
Denaturation	98°C	10 s	N cycles
Annealing	63°C	30 s	
Extension	72°C	20 s	
Hold	10°C	forever

**CRITICAL:** Typically, the number N is between 6 and 14, i.e., the total number of cycles needed is between 10 and 18 cycles depending on the number of cells and the abundance of the protein. See Troubleshooting 2.

28. Purify the library PCR product using 1.2× beads ratio using AmpureXP for PCR Purification beads or VAHTS DNA Clean Beads, according to manufacturer’s instructions. Elute the library using 30 μL Elution Buffer from the Qiagen minElute PCR Purification kit or 10 mM Tris-HCl, pH 8.0.

## Expected Outcomes

**Figure 8:**
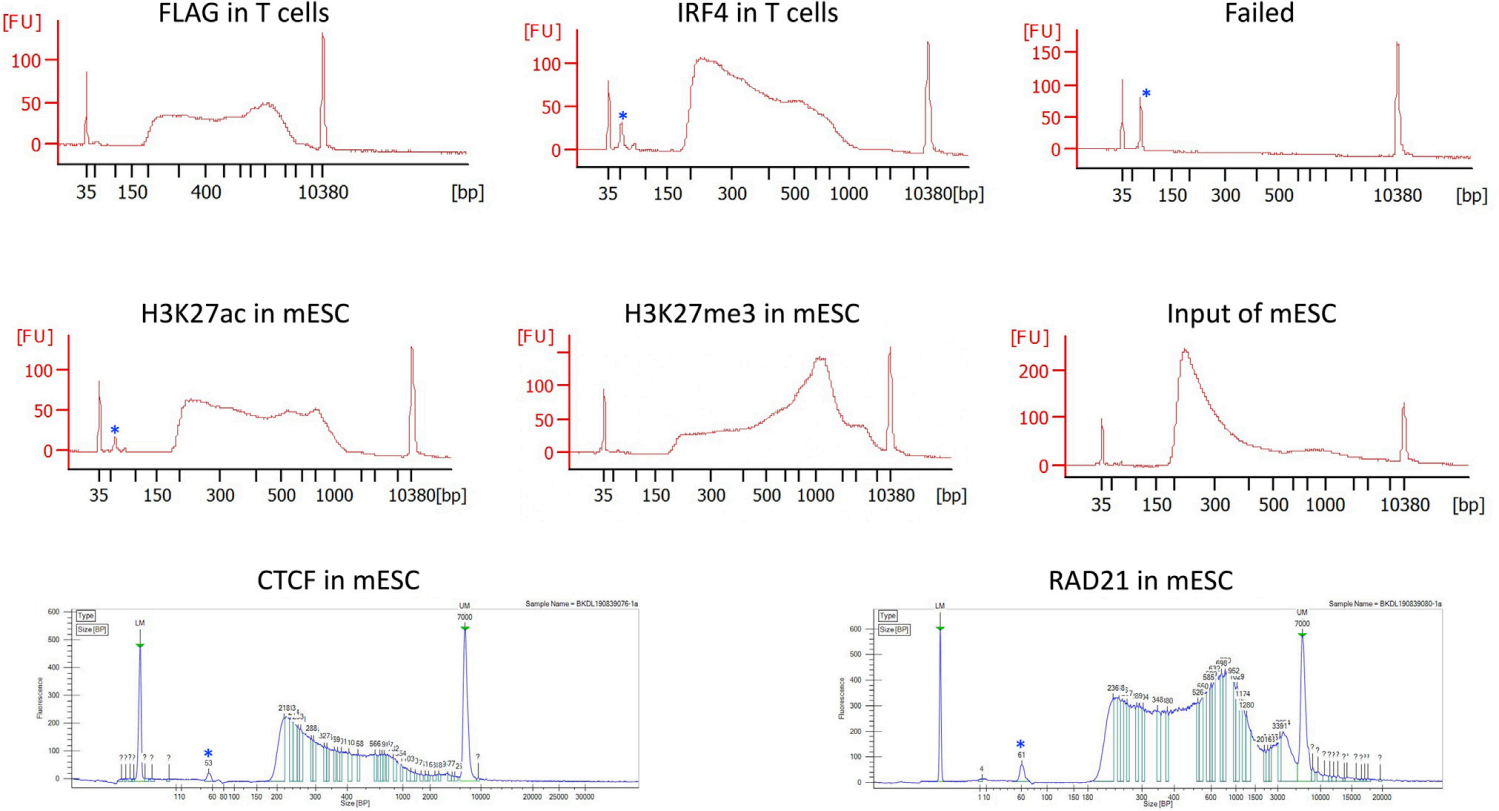
Examples of Successful and Failed Libraries on an Agilent Bioanalyzer 2100 and Caliper LabChip GX Asterisks indicate primer leftover.

The quality and quantity of the purified library should be checked by an Agilent Bioanalyzer 2100 machine or the like. We use the Agilent High Sensitivity DNA Kit and follow exactly the steps described in the manufacturer’s manual. Figure 8 shows a few examples of successful libraries and a failed one in different machines. One should expect at least 4 nM at the region between 200 bp and 1,000 bp.

***Note:*** The shape of the size distribution of the library depends on many factors, such as the sonication and the protein of being analyzed. The majority of the DNA should fall between 200 and 1,000 bp. We found the large fragments (>1,000 bp) do not affect quantification or sequencing at all. Therefore, we just leave them as they are. Asterisks indicate primer leftover, which can be removed by a further beads purification if needed.

If the libraries look good, send for sequencing. We normally perform 50 bp pair end sequencing, but single end sequencing can also be used. We followed the ENCODE ChIP-seq guide (Landt et al., 2012) to sequence at least 20 million reads for each experiment, which is usually enough for point source factors such as many transcription factors. For other factors that have broad binding patterns, such as H3K27me3 and H3K9me3, deeper sequencing is needed.

## Quantification and Statistical Analysis

Unfortunately, a successful library preparation does not mean a successful ChIPmentation experiment. Some preliminary computational analysis on the data need to be performed to see if the ChIPmentation experiment is working or not.

### Sequence Read Alignment

**Timing: 10 min to hours depending on computing power and sequencing depth**

Use the following command for the read alignment and format conversion:

hisat2 -p {threads} --no-temp-splicesite --no-spliced-alignment -x {genome} -1 {read1.fq.gz} -2 {read2.fq.gz} --summary-file mapping_stats.txt | samtools view -@ {threads} -ShuF 4 -q 30 - | samtools sort - -T {factor}_tmp -o {factor}_q30_sorted.bam

***Note:*** The above command is in one single line. Change {threads} to the number of cores you want to use, {genome} to the hisat2 genome index, {read1.fq.gz} and {read2.fq.gz} to your sequencing read file names, {factor} to the name of the factor that is being investigated. The commands align the reads to the genome, remove un-aligned reads, sort the reads by coordinates and only keep reads with mapping quality higher than 30.

### Peak Calling

**Timing: at least 10 min depending on sequencing depth**

Take the output bam file from the Sequence Read Alignment step, and use the following command to identify the genomic binding sites of the factor of interest:

macs2 -t {factor}_q30_sorted.bam -c input_q30_sorted.bam -g {genome_size} -f BAMPE -q 0.01 -B --SPMR -n {factor}

***Note:*** The above command is in one single line. Change {factor} to the name of the factor that is being investigated, and {genome_size} to the appropriate genome size or species code. Check the MACS2 manual for more information.

There will be a file in bedGraph format called {factor}_treat_pileup.bdg generated after the MACS2 peak calling. It is recommended to convert it to the bigWig format for the visualization. First, get the chromosome sizes for the genome, using the human genome hg38 as an example:

fetchChromSizes hg38 > hg38.chrom.sizes

Then, use the following command for the conversion:

bdg2bw {factor}_treat_pileup.bdg hg38.chrom.sizes

***Note:*** For more information about different file formats, check the UCSC genome browser documentation: https://www.genome.ucsc.edu/FAQ/FAQformat.html

### Assessment of Results

It is difficult to define universal rules to check whether a ChIPmentation experiment works or not. In our experience, the peak file generated during the peak calling process by MACS2 should contain thousands or even tens of thousands of peaks when an experiment is successful. However, some factors may have very few binding peaks. We have found visual inspection of the binding signal in the bigWig file using UCSC genome browser can be very helpful. See Troubleshooting 3.

**Figure 9:**
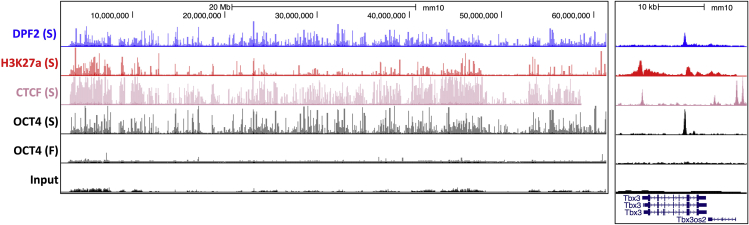
UCSC Genome Browser Screenshot of ChIPmentation Examples Both successful (S) and possibly failed (F) experiments are shown. On the left, the entire mouse chromosome 19 is shown. On the right, the locus of the Tbx3 gene is displayed.

First, look at the chromosome-wide view of the experiment. For successful experiments, clear “spikes” should be apparently visible to eyes, and there should be many comparing to the input sample. For failed experiments, it is relatively flat. When zooming in into specific target genes, you should see smooth bell-curve shaped peaks. The peak and the background can be easily discriminated by eyes. See examples in Figure 9.

## Limitations

The nature of ChIPmentation is essentially ChIP-seq. Many limitations that restrict the use of ChIP-seq also apply to ChIPmentation. The technique still requires a ChIP-grade antibody that can recognize and pull down its target protein after formaldehyde crosslinking. In general, finding a ChIP-grade antibody is difficult and time consuming. Alternatively, stable cell line expressing an epitope tagged version of the protein of interest can be generated, and an antibody against the tag can be used for ChIPmentation. For example, the ENCODE project has used the 3xFLAG tag to investigate genomic locations of hundreds of chromatin-associated proteins (Partridge et al., 2020), but this cannot be achieved in primary cells and tissues.

In addition, ChIPmentation only simplifies the procedure and increase the sensitivity of the library preparation steps. It does not change the chromatin immunoprecipitation part of the protocol. Therefore, the cell number required for a ChIPmentation experiment is relatively low comparing to the traditional ChIP-seq methods. In our hands, the minimum cell number for a successful ChIPmentation experiment is 10^4^ for profiling histone modifications and 10^5^ for investigating transcription factors. However, these numbers are still high and prohibit the profiling of rare material. If cell number is very limited, other methods such as uliChIP-seq (Brind’Amour et al., 2015), CUT&RUN (Skene and Henikoff, 2017), STAR ChIP (Zhang et al., 2016), CUT&TAG (Kaya-Okur et al., 2019), ACT-seq (Carter et al., 2019), itChIP-seq (Ai et al., 2019) can be used.

## Troubleshooting

### Problem 1

Poor sonication results: the majority of fragments are too large (>1 kb), or too small (100–200 bp) or heterogeneous (the presence of both large and small fragments at the same time), or not enough input DNA to visualize on agarose gels due to low number of cells.

### Potential Solution

When either large fragments or small fragments are present, adjust the sonication condition accordingly. The most straightforward approach is to change the number of cycles of the sonication, but sometimes, one needs to change the ON/OFF time. For histone modification, small fragments may not be a big problem.

We have found heterogeneous sonication often results from heterogeneous crosslinking. This often happens for cells that grow in colonies (i.e., not monolayer) or for primary cells not properly dissociated from tissues. Optimize your system to get good single-cell suspension first (i.e., use trypsin, collagenase etc.), and start from step 1 of the Step-By-Step Method Details section to crosslink the cells in solution.

If cells number is limited, and not enough DNA is recovered, one can reverse crosslink and purify DNA from the entire sample (instead of taking a fraction out).

### Problem 2

Low yield of immunoprecipitated DNA: this can be reflected at the qPCR stage where N (is step 26) is high (>14) or a flat profile on Bioanalyzer or the like.

### Potential Solution

It should be noted that high number of cycles does not necessarily mean a failed experiment, but a flat profile indicates the experiment probably failed. This could be due to low abundance of the protein of interest, or low antibody affinity to the protein. Increase the starting number of cells to see if it helps. In addition, trying different antibodies or adding an epitope tag to the protein of interest often help. Commonly-used tags with good antibodies include 3xFLAG, V5, and 3xHA. See the ENCODE ChIP-seq guide (Landt et al., 2012) for more details.

### Problem 3

Library preparation is successful, but the sequencing results suggest low signal-to-noise ratio. This is the most frequently encountered problem according to our experience.

### Potential Solution

Like suggested in the previous section, a successful library does not necessarily mean a successful ChIPmentation. Include a positive control antibody, such as a transcription factor antibody that has been tested successfully in ChIP-seq/ChIPmentation by neighboring labs to make sure the protocol is working as intended. If the positive control works, but the actual experiment fails, try to change to a different antibody or considering adding tags to the protein of interest.

Another reason could be the protein of interest does not interact with DNA tightly or directly, a dual crosslinking step can be used. Formaldehyde can crosslink both protein-DNA and protein-protein interactions, but it has poor efficiency of crosslinking protein-protein interactions due to its short spacer arm. A protein-protein crosslinker with a longer spacer arm (such as EGS) can be used to secure protein-protein interaction first, then formaldehyde is used to crosslink protein-DNA interaction. Check (Zeng et al., 2006) for details.

Finally, the right crosslinking condition also needs to be tested. If under-crosslinking happens, the protein will not be efficiently crosslinked to DNA and the bound DNA may be lost during the washes. If over-crosslinking happens, the protein epitope will be destroyed and the antibody will not be able to recognize the protein of interest. Both cases result in low signal-to-noise ratio. We suggest choosing a time course of crosslinking and perform the ChIPmentation experiment in parallel to find the condition that gives the best signal-to-noise ratio.

## Resource Availability

### Lead Contact

Further information and requests for resources and reagents should be directed to and will be fulfilled by the Lead Contact, Xi Chen (chenx9@sustech.edu.cn).

### Materials Availability

This study did not generate new unique reagents. All materials used in this study are commercially available, and the detailed information can be found in the Key Resources Table.

### Data and Code Availability

This study did not generate new data or code.
